# *Bacopa monnieri* Phytochemicals Mediated Synthesis of Platinum Nanoparticles and Its Neurorescue Effect on 1-Methyl 4-Phenyl 1,2,3,6 Tetrahydropyridine-Induced Experimental Parkinsonism in Zebrafish

**DOI:** 10.1155/2013/972391

**Published:** 2013-03-04

**Authors:** Jayshree Nellore, Cynthia Pauline, Kanchana Amarnath

**Affiliations:** ^1^Department of Biotechnology, Sathyabama University, Jeppiaar Nagar, Rajiv Gandhi Salai Chennai-119, Chennai, Tamilnadu, India; ^2^Department of Biochemistry, Sathyabama University, Chennai 600119, Tamilnadu, India

## Abstract

Current discovery demonstrates the rapid formation of platinum nanoparticles using leaf extract of a neurobeneficial plant, *Bacopa monnieri* (BmE). The nanoparticles (BmE-PtNPs) were stabilized and then coated with varied phytochemicals present within the leaf extract. These nanoparticles demonstrated the same activity of Complex I, as that of oxidizing NADH to NAD^+^ using a spectrophotometric method. This suggests that BmE-PtNPs are a potential medicinal substance for oxidative stress mediated disease with suppressed mitochondrial complex I, namely, Parkinson's disease (PD). Hence, the neuroprotective potentials of the phytochemical coated nanoparticle were explored in 1-methyl 4-phenyl 1,2,3,6 tetrahydropyridine- (MPTP-)induced experimental Parkinsonism in zebrafish model. BmE-PtNPs pretreatment significantly reversed toxic effects of MPTP by increasing the levels of dopamine, its metabolites, GSH and activities of GPx, catalase, SOD and complex I, and reducing levels of MDA along with enhanced locomotor activity. Taken together, these findings suggest that BmE-PtNPs have protective effect in MPTP-induced neurotoxicity in this model of Parkinson's disease via their dual functions as mitochondrial complex I and antioxidant activity.

## 1. **Introduction**

Parkinson's disease (PD) is a neurodegenerative disease of dopamine (DA) neurons in substantia nigra characterized predominantly by resting tremors, bradykinesia, muscular rigidity, and postural instability, along with several nonmotor symptoms [1]. The disease is associated with a loss of antioxidants or increase in prooxidant levels and mitochondrial dysfunction. The neurotoxin 1-methyl-4-phenyl-1,2,3,6- tetrahydropyridine (MPTP) is known to cause a similar loss of dopaminergic neurons in the human midbrain with corresponding Parkinsonian symptoms [2]. Several animal species have also shown sensitivity to MPTP, including primates, mice, goldfish, and, most recently, zebrafish [3]. MPTP is metabolized to 1-methyl-4-phenyl pyridinium (MPP^+^) in glial cells in the brain. After release from the glia, MPP^+^ is transported into dopaminergic neurons via the dopamine transporter (DAT). MPP^+^ accumulates in the mitochondria and induce neuronal cell death via several pathways, including the inhibition of complex I activity of the respiratory chain. This contributes to both reactive oxygen species generation and nigral cell loss. [4]. The excessive production of reactive oxygen species, such as superoxide anion, hydroxyl radical and hydrogen peroxide, may either directly damage the cellular macromolecule to cause cell necrosis or affect normal cellular signaling pathways and gene regulation to induce apoptosis [5]. 

Lately, several studies demonstrated the free radical scavenging activity, reducing the concentration of reactive oxygen, and nitrogen species of artificial antioxidants include inorganic nanoparticles possessing intrinsic antioxidant properties and nanoparticles functionalized with natural antioxidants or antioxidant enzymes [6]. 

Recent studies have proposed that platinum in the form of nanoparticles has an activity that is similar to that of oxidizing Nicotinamide adenine dinucleotide (NADH) and reducing ubiquinone (CoQ_10_) [7]. Platinum nanoparticles also provide dual functions as mitochondrial complex I to lower reactive oxygen species (ROS) generation and as *Superoxide* dismutases (SOD)/catalase mimetics to scavenge ROS including superoxide anion (O^2−^) as well as hydrogen peroxide (H_2_O_2_) and free radicals [8, 9]. This suggests that platinum nanoparticles can mimic a part of the enzymatic functions of the complex I and indicates their possible use in medical treatments for Parkinson's disease.

With such important applications, it is imperative to develop platinum nanoparticles through environmentally sound and nonpolluting technologies. While a number of chemical methods [7] are currently available and are extensively used, the collaborations are often energy intensive and employ toxic chemicals, thereby precluding biomedical application. With the flourishing demand on “green” nanotechnological processes [10], the field of nanoparticle synthesis has recently developed new routes. Biosynthetic methods employing either biological microorganisms [11] or plant extracts [12] have emerged as simple and viable alternative to chemical synthetic procedures. Using plants for nanoparticle synthesis can be advantageous over other biological processes, because they eliminate the elaborate process of maintaining cell cultures and can also be suitably scaled up for large-scale nanoparticle synthesis [13].

 In this work presented here, we describe a single-step “green synthesis” protocol for the production of well-defined platinum nanoparticles by utilising aqueous extract of *Bacopa monnieri* (BM) leaves. We have chosen *Bacopa monnieri* (BM) leaves because it is often used to treat people with Parkinson's disease [14]. Studies suggest that they improve circulation to the brain, as well as improving mood, cognitive function, and general neurological function [15]. The presence of bacoside A and B in BM leaves attributes to their neurobeneficial function [16]. In addition to the neurobeneficial effects they exert antiamnesic [17], antioxidant [18], antistress [19], anxiolytic [20], memory enhancing [21], and antiulcerogenic activities [19]. Recent studies suggests that *Bacopa monnieri* also exerts antiinflammatory [22] and antiarthritis activity [23]. Hosamani and Muralidhara have reported the neuroprotective efficacy of *Bacopa monnieri* against rotenone-induced oxidative stress and neurotoxicity in *Drosophila melanogaster *[24]. Recent studies have demonstrated that pretreatment with the BM extract protected the human neuroblastoma cell line SK-N-SH against H_2_O_2_ and acrolein by modulating the activity of several redox regulated proteins, that is, NF-kappaB, Sirt1, ERK1/2, and p66Shc, so as to favor cell survival in response to oxidative stress [25]. Based on these stipulations, we used BM extract in the present study to prove its synergistic reduction potential in reducing platinum salts and for the creation of robust coating on platinum nanoparticles to produce stable phyto platinum nanoparticles for potential applications in medicine and technology. Since recent research in antioxidant nanomaterial has opened a new era in pharmaceutical industries, we, hence forth finally, propose to determine the potentiality of platinum nanoparticles coated with phytochemicals of *Bacopa monnieri* leaf (BmE-PtNPs) extract endowed with antioxidants to mitigate the oxidative damage induced by MPTP in zebrafish (Danio rerio). 

Thereby, in the present, we aimed to explore the neuroprotective role of BmE-PtNPs in MPTP-induced zebrafish PD model.

## 2. Experimental Procedure

### 2.1. Materials

The components (natural constructs) used in the synthesis of platinum nanoparticles (PtNPs) were procured from standard vendors. MPTP and Chloroplatinic acid (H_2_PtCl_6_) was purchased from Bio Corporals, Vadapalani, Chennai. Nicotinamide adenine dinucleotide (NADH), 5, 5′-dithio-bis (2 nitro benzoic acid) (DTNB), nitro blue tetrazolium, perchloric acid, potassium dihydrogen phosphate, Acetonitrile, citric acid, 5-5′-dithiobis-p-nitrobenzoic acid, Potassium Dihydrogen Phosphate (KH_2_PO_4_), Ethylene diamine tetra acetic acid (EDTA), and octanesulfonic acid were obtained from Southern Scientific Corporation, Chennai.

#### 2.1.1. Synthesis of *Bacopa monnieri*-Stabilized Platinum Nanoparticles (BmE-PtNPs)

A general method was used to synthesize platinum nanoparticles with slight modifications [12]. The *Bacopa monnieri* leaves extract (BmE) was prepared by weighing 50 g of *Bacopa monnieri* leaves in 250 mL beaker along with 100 mL of distilled water and maintained at 80°C for 2 hrs before decanting it. The solution was filtered by 0.45 *μ*m Milipore membrane filter and followed by 0.2 *μ*m Millipore membrane filter. For the synthesis of platinum nanoparticles, 40 ml of 1 mM was reacted with 10 mL of the *Bacopa monnieri* leaves extract in Erlenmeyer flask at room temperature. The formation of platinum nanoparticles was confirmed by the change in the color of the mixture from pale yellow color to dark brown.

The final concentration of metal nanoparticles in the solution was around 6 × 10^−4^ M. The NPs synthesized were found to be stable for more than a month when stored in closed containers, and no visible change was observed for several days.

#### 2.1.2. Characterization

The characterization of synthesized nanoparticles was carried out according to the methods described previously [26]. The stability and identity of the BmE-PtNPs were measured by recording UV absorbance. The absorbance peak at ~330–380 nm confirmed the retention of nanoparticulates in all the above mixtures. The size and shape of the nanoparticles were determined by using TEM on a JEOL TEMSCAN2000EX model operating at accelerating voltage at 80 KeV. The sample for TEM was prepared by putting one drop of the suspension onto standard carbon-coated copper grids and then drying under an IR lamp for 30 min. FTIR spectra of freeze-dried BmE-PtNPs were investigated by analyzing the sample under Brukere Tensor 27 FTIR spectrometer in attenuated total reflection mode using the spectral range of 2000–400 cm^−1^ with the resolution of 4 cm^−1^. The energy dispersive X-ray analysis of isolated nanoparticles was carried out by means of JEOL EDX model-JSM-5610 LV.

#### 2.1.3. Oxidation of NADH by BmE-PtNPs

To investigate the chemical change of BmE-PtNPs by NADH oxidation, UV-Vis surface Plasmon resonance absorption spectra of BmE-PtNPs were measured from 200 to 800 nm after incubation with NADH. BmE-PtNPs at 50 *μ*g/mL were incubated with 100 *μ*M NADH in water at room temperature for 12 h. Subsequently, the incubated mixture was centrifuged to remove NADH and NAD which impede the spectrum measurement. Then, samples were redispersed with the equal volume of water. This washing process was repeated 10 times.

#### 2.1.4. Animals

Wild-type adult (<8 months old) zebrafish were obtained from specialized supplier. Animals were kept in aged tap water at 28°C under a 14 : 10 h light : dark cycle. Feeding and maintenance of fish were done according to Westerfield (1995) [27]. Animals were acclimated for at least 2 weeks before the experiments. The procedures were approved by the institutional animal ethics committee. 

#### 2.1.5. Experimental Groups

The experimental groups used for the tests comprise the following. (a) Experimental Parkinsonism-induced group (a single dose of MPTP (225 mg/kg bwt) injected intraperitoneally (I.P)) this dosage was based on a previous study that has demonstrated impaired locomotor activity in adult zebrafish [28] BmE-PtNPs -control group (various concentrations such 0.3 *μ*mol, 0.4 *μ*mol, and 0.5 *μ*mol/kg body weight suspended in physiological saline (PS) were administered once in alternative days for 5 days). (b) BmE-PtNPs pretreated group (BmE-PtNPs + MPTP), various concentrations of BmE-PtNPs were administered once in alternative days for 5 days while MPTP was given on the 4th day 24 hours after the injection of BmE-PtNPs. (c) Control group (PS-injected but otherwise identically treated fish served as control group): the injections were conducted using Hamilton syringes with a mean injection volume of 5 *μ*L/g body weight. The study was carried out in group of 6–8 fish per treatment.

The fish were sacrificed after MPTP injections to observe the effects of BmE-PtNPs, 24 hours for lipid peroxidation, the activities of antioxidant systems and complex I and 5 days for the locomotor activity, content of dopamine and its metabolites in zebrafish brain. 

### 2.2. Preparation of Brain Homogenates

The whole brain tissue homogenates were prepared in 0.1 M phosphate buffer and centrifuged at 3000 × g for 30 min. The supernatants were used in the experiments. Protein concentration was estimated by Lowry's method [29]. 

### 2.3. MDA, GSH, SOD, GSH-Px, Catalase, and Total Antioxidant Capability Assay

The brain supernatants were then subjected to the measurement of malondialdehyde (MDA), glutathione (GSH), superoxide dismutase (SOD), glutathione peroxidase (GSH-Px), catalase (CAT), and total antioxidant capability by spectrophotometric methods. The Thiobarbituric Acid Reactive Substance (TBARS) was measured to analyse the MDA levels [30]. GSH content was measured according to the method of [31] based on the reacting with 5, 5′-dithio-bis (2 nitro benzoic acid) (DTNB or Ellman's reagent) to give a yellow colour compound that absorbs at 412 nm. The activity of SOD was assayed by monitoring the inhibition of the reduction of nitro blue tetrazolium by the sample at 560 nm [32]. Catalase was analysed as the rate of decrease in absorbance of H_2_O_2_ at 240 nm/min/mg protein [33]. Glutathione peroxidase was detected with 5-5′-dithiobis-p-nitrobenzoic acid [34]. 

### 2.4. Estimation of Complex I Activity

Complex I activity in crude mitochondrial preparation from zebrafish whole brain was monitored by the decrease in absorbance at 340 nm due to the oxidation of NADH [35]. 

#### 2.4.1. Catecholamine Measurements

The contents of dopamine and its metabolites were determined according to the method of Luo et al., 2009 [36]. Briefly, whole brain tissue was homogenised in 0.5 mL of cold perchloric acid (0.4 M). Subsequently, the sample was centrifuged at 20,000 × g for 10 min at 4°C, and the supernatant was transferred to a clean tube and measured for volume. One-half volume of a solution containing 0.02 M potassium citrate, 0.3 M potassium dihydrogen phosphate, and 0.002 M Na_2_EDTA was added and mixed thoroughly to deposit perchloric acid. After incubation in an ice bath for 60 min, the mix was centrifuged at 15,000 g, for 20 min at 4°C. Supernatants were analyzed for catecholamines, especially dopamine and its metabolite 3.4-dihydroxyphenylacetic acid (DOPAC), by HPLC (125 mm × 3 mm I.D. column, packed with Nucleosil 100 C 18; 3 *μ*m particle size) and electrochemical detection (INTRO, ANTEC Leyden, The Netherlands; cell potential = 800 mV). The mobile phase consisted of 5% acetonitrile, 10 g/L citric acid, 4 g/L KH_2_PO_4_, 0,1 g/L EDTA, and 0,175 g/L octanesulfonic acid; pH = 3.0. 

#### 2.4.2. Locomotor Activity Assessment

The locomotor activity of zebrafish was measured as per the protocol followed by Xia et al., 2010 [37] with slight modifications. A small experimental tank (30 cm × 10 cm × 15 cm) contained 3 L water was used to assess the locomotor activity of zebrafish. A transparent plastic film was placed in front of the tank and divided the tank into four segments. Fish were placed individually in the tank and their behavior was video recorded for 5 min after a 10 min habituation period. Spontaneous swimming activity was measured by recording the distance traveled, mean speed, and number of times the subject moved from one section into another during a 5 min observation.

## 3. Results and Discussion

**Figure 1:**
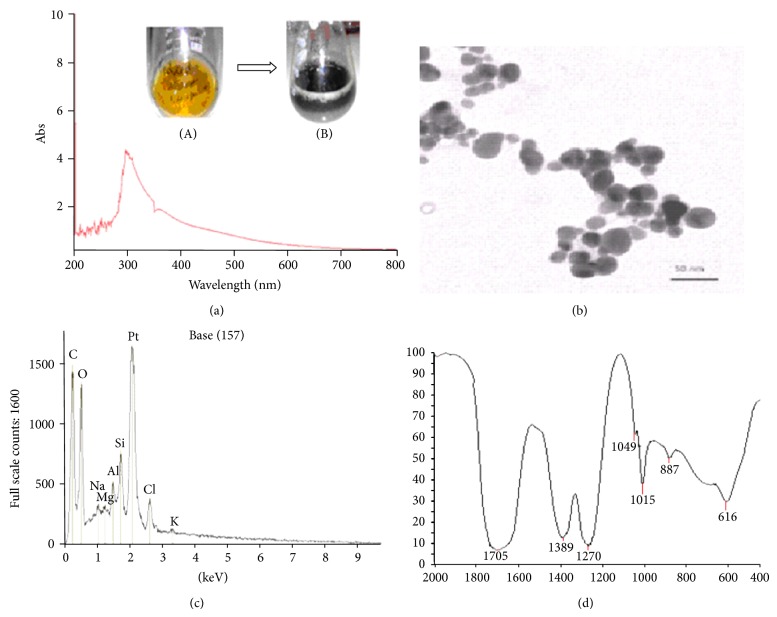
Characterization of *Bacopa monnieri* phytochemicals coated platinum nanoparticles (BmE-PtNPs). (a) UV-Vis Spectra of BmE-PtNPs. The inset shows two bottles with the *Bacopa monnieri* leaf extract before (A) and after (B) reaction with 1 mM PtCl_6_^2−^ ions for 3 hrs at 95°C. A color version of the inset can be seen. (b) TEM images of BmE-PtNPs. (c) EDAX of BmE-PtNPs. (d) FTIR spectra of BmE-PtNPs.

Our new process for the production of *Bacopa monnieri* phytochemicals coated platinum nanoparticles (BmE-PtNPs) uses a direct interaction of Chloroplatinic acid with *Bacopa monnieri* leaf extract in aqueous media without the intervention of any external man-made chemicals and, hence, 100% biogenic. The colloidal solution of BmE-PtNPs showed a very intense brown color which indicates the reduction of platinum ions (Figure 1(a) (inset)). The formation of BmE-PtNPs was further confirmed by tracing the reaction with UV-Visible spectroscopy. The absorption spectrum of the brown platinum collides prepared by biogenic process showed a surface plasmon absorption band with a maximum of ~340 nm (Figure 1(a)). TEM (Figure 1(b)) analysis exposes mostly spherical shaped platinum nanoparticles of approximate size of 5–20 nm. Under careful observation, it was evident that the edges of the particles were lighter than the centers, suggesting that some bioorganic compounds such as proteins in *Bacopa monnieri *leaf extract capped the platinum NPs contributing to excellent robustness against agglomeration [13]. The result obtained in the synthesis and characterization of nanoparticles is strongly supported by previously published report on the synthesis of platinum nanoparticles using phytochemicals [12]. The compositional analysis through energy dispersive X-ray (EDX) spectrometers illustrated the purity of the platinum, with the spectra showing a strong Pt signal (Figure 1(c)). Prominent bands were observed in the FTIR spectra (Figure 1(d)) at 616, 887, 1015, 1049, 1270, 1389, and 1705 cm^−1^, these peaks are assigned to alcohols C–N stretching vibration of aliphatic amines, phenolic groups, C–N stretching vibration of aromatic amines, germinal methyls, C=C groups or aromatic rings, and carbonyl groups, respectively. Significant peaks were not observed in the amide I (1640 cm^−1^) or amide II (1540 cm^−1^) regions that are characteristic of proteins/enzymes accountable for the reduction of metal ions to nanoparticles by biological processes. These results indicate that phytochemicals of BM leaf extract like flavanoids that have functional groups of amines, alcohols, ketones, aldehydes, and carboxylic acid are robustly coated over the platinum nanoparticles synthesized. Because of the phytochemical coating and the redox chemistry of BmE-PtNPs, it is possible that they are biologically active as antioxidants [13].

**Figure 2:**
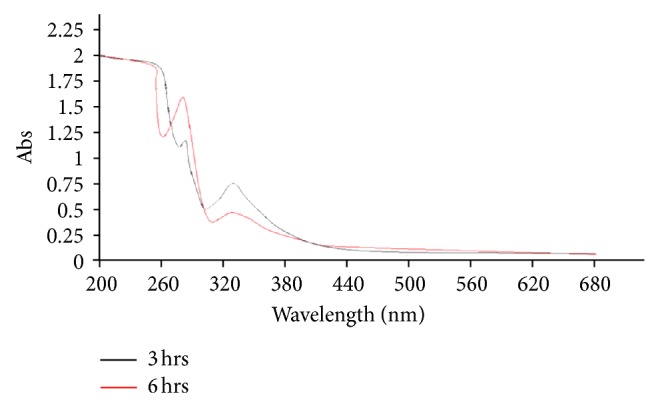
Change in absorption spectra at 3 h and 6 h, respectively. BmEPt nps were incubated with NADH in water at room temperature. The concentrations of platinum in BmE-PtNPs and NADH were 50 *μ*g/mL and 100 *μ*M, respectively.

To determine if BmE-PtNPs can oxidize NADH, 100 *μ*M NADH was incubated with 50 *μ*g/mL BmEPt nps for 3 h and 6 h, respectively. The absorbance decreased and increased with time at 340 and 260 nm, respectively (Figure 2). This observation indicated that BmE-PtNPs oxidized.

*NADH* to *NAD* ^+^. This is because the bands at 340 and 260 nm are from the *n*-*π*∗ transition of dihydronicotinamide part and *π*∗-*π*∗ transition of the adenine ring, respectively. This result demonstrates that BmE-PtNPs have an activity similar to mitochondrial NADH : Ubiquinone oxidoreductase, which is concurrence with the earlier published results of pectin protected platinum nanoparticles [9]. This suggests that BmE-PtNPs are a potential medicinal substance for oxidative stress mediated disease with suppressed mitochondrial complex I, namely, Parkinson's disease (PD).

**Figure 3:**
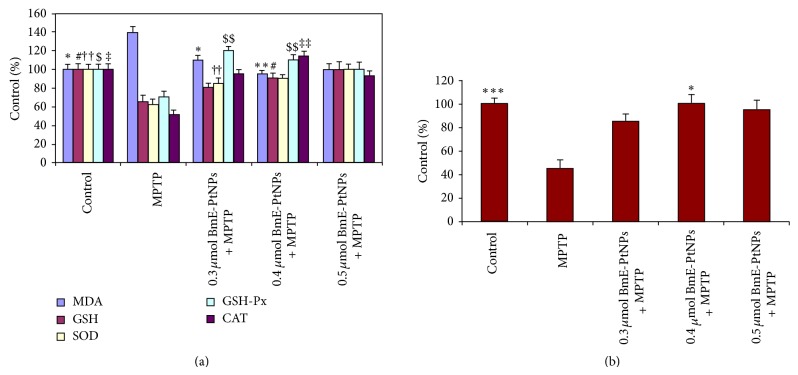
(a) Effects of *Bacopa monnieri* phytochemicals coated platinum nanoparticles (BmE-PtNPs) on the content/activity of MDA, GSH, SOD, GSH-Px, and CAT in the MPTP zebrafish brain. Data were shown as mean ± SEM. *n* = 6–8, ^∗#†$‡^ *P* < 0.05, ^∗∗##††$$‡‡^ *P* < 0.01, ^∗∗∗###†††$$$‡‡‡^ *P* < 0.001 versus MPTP group. (b) Effects of *Bacopa monnieri* phytochemicals coated platinum nanoparticles (BmE-PtNPs) on the activity of complex I in the MPTP zebrafish brain. Data were shown as mean ± SEM. *n* = 6–8, ^*^ *P* < 0.05, ^**^ *P* < 0.01, ^***^ *P* < 0.001 versus MPTP group.

Oxidative stress was generated in zebrafish by exposure to MPTP, which is an intracellular free radical-generating compound resulting in corresponding Parkinsonian symptoms [2]. The intraperitoneal administration of a single dose of MPTP (225 mg/kg bwt) resulted in a profound increase in the levels of MDA, diminished activities of antioxidant defense mechanism in charge for scavenging free radicals and maintaining redox homeostasis such as SOD, CAT, GPx, GSH, and complex I were observed in experimental Parkinsonism-induced group (MPTP) (Figures 3(a) and 3(b)). The BmE-PtNPs concentrations tested were 0.3, 0.4, and 0.5 *μ*mol, respectively. The MDA levels were significantly decreased by 0.4 *μ*mol of BmE-PtNPs (Figure 3(a)). This makes clear the inhibitory effect of BmE-PtNPs over ROS generation during MPTP-induced oxidative stress.

Glutathione (GSH) is a tripeptide with a free reductive thiol functional group, responsible for the detoxification of peroxides such as hydrogen peroxide or lipid peroxides and acting as an important antioxidant in cells. During the detoxification process, GSH (reduced form) becomes oxidized glutathione (GSSG) which is then recycled to GSH by the enzyme glutathione reductase present in cells. The increased MDA levels in MPTP could be due to their increased production and/or decreased destruction by antioxidants such as GSH, SOD, catalase, and glutathione peroxidase [38]. The activities of antioxidant defense enzymes in charge for scavenging free radicals and maintaining redox homeostasis such as GSH, SOD, catalase, and glutathione peroxidase are diminished during oxidative stress induced by MPTP. In the present study, a statistically significant increase in the levels of GSH, SOD, catalase, and glutathione peroxidase in the MPTP treated zebrafish with 0.4 *μ*mol of BmE-PtNPs is being proved. This study demonstrates that BmE-PtNPs act as reductive catalyst, by the ability to scavenge ROS, superoxide anion radicals (O_2_^−^), and hydrogen peroxide (H_2_O_2_). This data is in good accordance with the previous studies where platinum nanoparticles are known to act as a SOD/catalase mimetics to extend the lifespan of C. elegans, extended the lifespan of the roundworm Caenorhabditis elegans[39–41], inhibited pulmonary inflammation in mice exposed to cigarette smoke [42], inhibited cell growth of human tongue carcinoma cells [43], and furthermore ameliorated neurological function and brain damage after ischemic stroke [44].

The mitochondrial respiratory chain, especially at complexes I, is thought of as a primary site of ROS generation. In some oxidative stress diseases such as Parkinson's disease, excessive ROS generation is responsible for pathogenesis due to the suppression of complex I. In the current study a significant inhibition of complex I activity was observed in the experimental Parkinsonism-induced group (Figure 3(b)), which was attenuated by the pretreatment of various concentrations of BmE-PtNPs (Figure 3(b)). However, 0.4 *μ*mol of BmE-PtNPs demonstrated a noteworthy effect on restoring the complex I activity as well as the levels of GSH, SOD, catalase, and glutathione peroxidase in the MPTP treated zebrafish. This result demonstrates that BmE-PtNPs serve dual functions as mitochondrial complex I to lower ROS generation and as SOD/catalase mimetics to scavenge generated excessive ROS.

**Figure 4:**
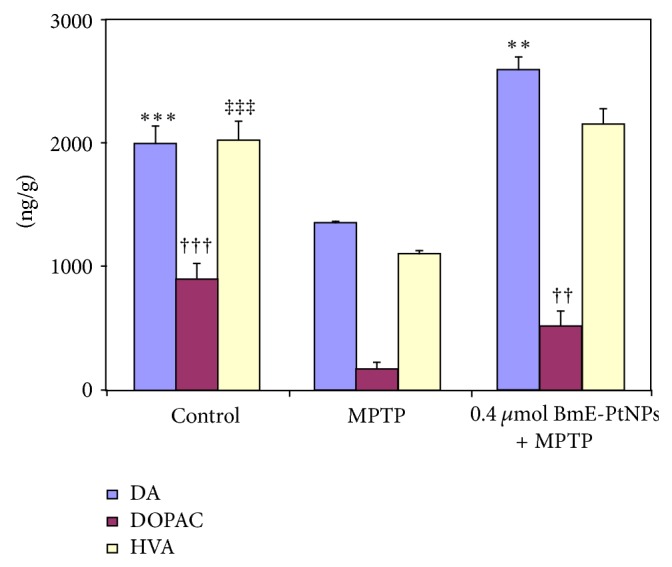
Effects of *Bacopa monnieri* phytochemicals coated platinum nanoparticles (BmE-PtNPs) on the contents of dopamine, DOPAC, and HVA in the MPTP zebrafish brain. Data were shown as mean + SEM. *n* = 6–8, ^∗†‡^ *P* < 0.05, ^∗∗††‡‡^ *P* < 0.01, ^∗∗∗†††‡‡‡^ *P* < 0.001 versus MPTP group.

Postmortem studies provided evidence for the decrease in the content of dopamine (DA) and its metabolites dihydroxyphenylacetic acid (DOPAC), and homovanillic acid (HVA) in the brains of Parkinson's disease. Our results showed that the DA, DOPAC, and HVA contents in MPTP zebrafish were markedly lower than those of control fish, and BmE-PtNPs increased DA, DOPAC, and HVA levels (Figure 4). 

**Figure 5:**
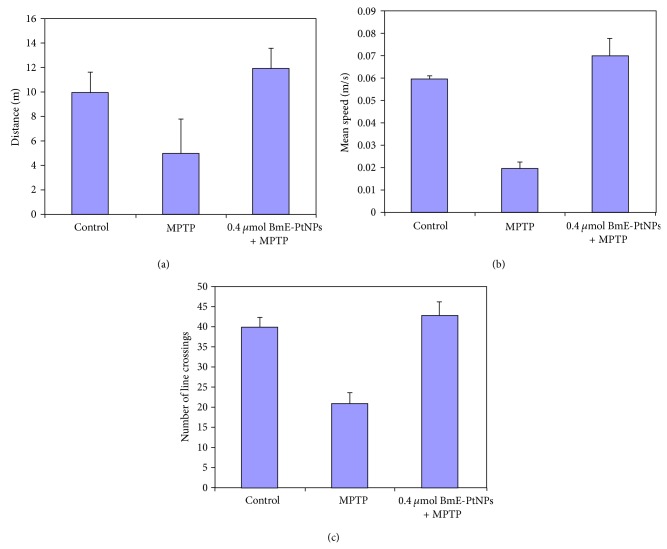
Effects of *Bacopa monnieri* phytochemicals coated platinum nanoparticles (BmE-PtNPs) on zebrafish brain locomotor activity. Data were shown as mean ± SEM. *n* = 6–8; one-way ANOVA test was performed.

In Parkinson's disease, the most debilitating symptom of the disease is the loss of motor control.  Figure 5 shows the results for the locomotion activity. MPTP administration results in a significant reduction in the total movement distance, mean velocity, and mean distance per movement in zebrafish compared to the control animals. This finding points the correlate loss of dopamine due to MPTP neurotoxicity. However, these reductions were partially rescued in BmE-PtNPs (0.4 *μ*mol/kg body weight) treatment animals, while BmE-PtNPs alone did not affect the locomotor activity (data not shown). Our current results suggest that BmE-PtNPs may be potentially effective in protecting against ROS-mediated disease, by scavenging ROS under pathophysiological conditions. 

## 4. Conclusion

In conclusion, platinum nanoparticles (BmE-PtNPs) have been synthesized from *Bacopa monnieri *leaf extract (BmE). The analysis of the chemical composition by EDAX strongly suggests the formation of elemental platinum nanoparticles instead of their oxides. From the TEM analysis, the approximate sizes of the nanoparticles are found to be 5–20 nm. FTIR measurements provided strong evidence for proteins to form a coat covering the platinum nanoparticles to stabilize and prevent the agglomeration of the particles. This simple procedure for the biosynthesis of platinum nanoparticles has several advantages such as cost effectiveness, compatibility, and eco friendliness for biomedical and pharmaceutical applications. In addition, the ecofriendly method will be a competitive alternative to the existing methods for producing nanoscale inorganic materials.

The antioxidant and neurorescue activities of nanosized *Bacopa monnieri* phytochemicals coated platinum nanoparticles (BmE-PtNPs) were ascertained by alleviating the ROS generation and scavenging free radicals, thus increasing the levels of dopamine, its metabolites, GSH, and activities of GPx, catalase, SOD, and complex I and reducing levels of MDA along with enhanced locomotor activity. These results provide ample evidence pertaining to the neuroprotective ability of BmE-PtNPs in MPTP-induced neurotoxicity in zebrafish model of Parkinson's disease via its dual functions as mitochondrial complex I and antioxidant activity (SOD and catalase mimic activities). Further study is necessary to manifest the exact mechanism of action of BmE-PtNPs on neuroprotection in MPTP-induced experimental Parkinsonism in zebrafish model. Such a biocompatible and ecofriendly nanoparticle holds promise as a potential therapeutic option for Parkinson's disease.
